# Clinical utility of repeated IgH gene rearrangement testing for the diagnosis and surveillance of gastric MALT lymphoma

**DOI:** 10.1038/s41598-025-16512-5

**Published:** 2025-08-27

**Authors:** Hidehiko Takigawa, Yuki Kitadai, Daisuke Shimizu, Misa Ariyoshi, Takeshi Takasago, Akiyoshi Tsuboi, Hidenori Tanaka, Ken Yamashita, Yuichi Hiyama, Yoshihiro Kishida, Yuji Urabe, Akira Ishikawa, Ryo Yuge, Toshio Kuwai, Shiro Oka

**Affiliations:** 1 Department of Gastroenterology, Graduate School of Biomedical and Health Sciences, Hiroshima University, 1-2-3 Kasumi, Minami-ku, Hiroshima, Japan; 2 Clinical Research Center in Hiroshima, Hiroshima University Hospital, Hiroshima, Japan; 3 Department of Molecular Pathology, Graduate School of Biomedical and Health Sciences, Hiroshima University, Hiroshima, Japan; 4 Department of Gastroenterology, Hiroshima Red Cross Hospital and Atomic-bomb Survivors’ Hospital, Hiroshima, Japan; 5 Gastrointestinal Endoscopy and Medicine, Hiroshima University Hospital, Hiroshima, Japan

**Keywords:** IgH rearrangement, Pathological diagnosis, Gastric MALT lymphoma, Eradication therapy, Surveillance, Prediction, Gastrointestinal cancer, Gastric cancer, Cancer, Tumour biomarkers

## Abstract

Gastric MALT lymphoma diagnosis relies on histopathological findings and immunoglobulin H gene (IgHr) rearrangement testing, which reflects monoclonal immunoglobulin proliferation. This study aimed to clarify the role of IgHr in the diagnosis, treatment prediction, and surveillance of gastric MALT lymphoma. Of the 152 suspected cases, 131 were definitively diagnosed using a combination of IgHr and pathology, with pathological findings considered the gold standard. Patients with discrepancies between IgHr and pathology underwent re-evaluation. The relationship between IgHr status, clinicopathological features, and treatment outcomes was analyzed. IgHr and histopathology were assessed over 2 years in 41 patients after pathological complete remission (pCR). IgHr positivity was 69.5% at initial biopsy and 90.8% after two biopsies. IgHr-positive cases had higher *H. pylori* infection rates and better CR rates post-eradication. Patients with IgHr positivity at pCR had higher recurrence rates (16.7%). IgHr positivity gradually declined among 37 non-recurrent cases (CR: 56.8%, 6 M: 45.9%, 1Y: 21.6%, 2Y: 10.8%), indicating a delay between pCR and IgH-negative conversion. Repeated biopsies may improve the accuracy of gastric MALT lymphoma diagnosis. IgHr-positive status at pCR may signal higher recurrence risk, underscoring the need for careful post-CR surveillance. Surveillance should account for potential delays in IgHr-negative conversion.

## Introduction

Mucosa-associated lymphoid tissue (MALT) lymphoma is a low-grade B-cell lymphoma, with the stomach being the most prevalent site of gastrointestinal involvement. *Helicobacter pylori* (HP) infection and the BIRC3-MALT1 fusion gene are the primary causes of Gastric MALT lymphoma. However, non-*Helicobacter pylori helicobacters* (NHPH) infection has also recently been implicated as a causal factor in some cases^1,2^. MALT lymphoma is characterized by a noninvasive lymphoplasmacytic infiltrate^3^ and lymphoepithelial lesion (LEL)^4^. Immunohistochemistry typically shows CD3-negative, CD20-positive, and CD79a-positive staining^5,6^. Immunoglobulin H gene rearrangement testing (IgHr) to detect monoclonal B cell proliferation is diagnostically significant^7,8^. Combining histopathological findings and IgHr is useful for diagnosing gastric MALT lymphoma, but its diagnostic performance and behavior during surveillance remain unclear.

The standardized BIOMED-2 protocol enables sensitive detection of monoclonality in lymphomatous cells through multiplex polymerase chain reaction (PCR) analysis of IgHr^9^. A previous report with small sample sizes revealed a positive rate of IgHr in 6/12 gastric MALT lymphoma cases (50%)^10^. However, limited evidence exists from larger cohorts. Furthermore, a study on gastrointestinal MALT lymphoma reported that the positive rates of IgHr were 48.4% and 90.9% in Stage I/II and Stage III/IV cases, respectively^11^, suggesting that IgHr is partly negative in early-stage MALT lymphoma cases.

Conversely, Hsi et al. reported that IgHr could be detected in approximately 10% of cases with *HP*-associated gastritis, even in the absence of gastric MALT lymphomas^9^. Potential explanations for these false-positive results include non-neoplastic clonal B-cell proliferation secondary to infection or the detection of early lymphoma lesions that are not yet identifiable through histological examination. However, the precise mechanisms remain unclear.

Additionally, IgHr has been reported to persist even after pathological complete remission (CR) of MALT lymphoma^10^. This discordance between IgHr status and pathological findings can complicate the clinical assessment of CR and create challenges in determining CR status. Conversely, initial pathological examination in a case report of conjunctival MALT lymphoma showed no evidence of lymphoma; however, the IgHr was positive. Notably, MALT lymphoma was pathologically confirmed 11 months after repeated biopsies^11^. The perspectives on IgHr status change over time; however, comprehensive studies with substantial sample sizes are limited, leaving the longitudinal trends unclear.

This study analyzed the IgHr positivity rate at diagnosis, defining histopathological findings as the gold standard. For cases with discrepancies between pathological and IgHr results, a re-biopsy was performed to evaluate whether a repeat biopsy improves the IgHr-positive rate. Its role in diagnosis, predicting therapeutic efficacy, and surveillance of gastric MALT lymphoma was explored. Additionally, longitudinal changes in IgHr status and pathology were evaluated in patients with pathological CR to assess the clinical implications of IgHr in managing gastric MALT lymphoma.

## Methods

### Diagnostic flow for inclusion of patients with gastric MALT lymphoma

Between February 2013 and March 2023, 152 patients with endoscopically suspected gastric MALT lymphoma were retrospectively analyzed. All 152 patients underwent initial evaluation (histopathological examination with immunohistochemistry, IgHr, and BIRC3-MALT1 fusion gene analysis) using biopsy specimens from the lesion site.

Patients with positive IgHr and pathological results were diagnosed with gastric MALT lymphoma, while those with negative results in both examinations were defined as having no MALT lymphoma. Discrepant cases underwent re-biopsy and re-evaluation of both IgHr and pathology.

**Fig. 1:**
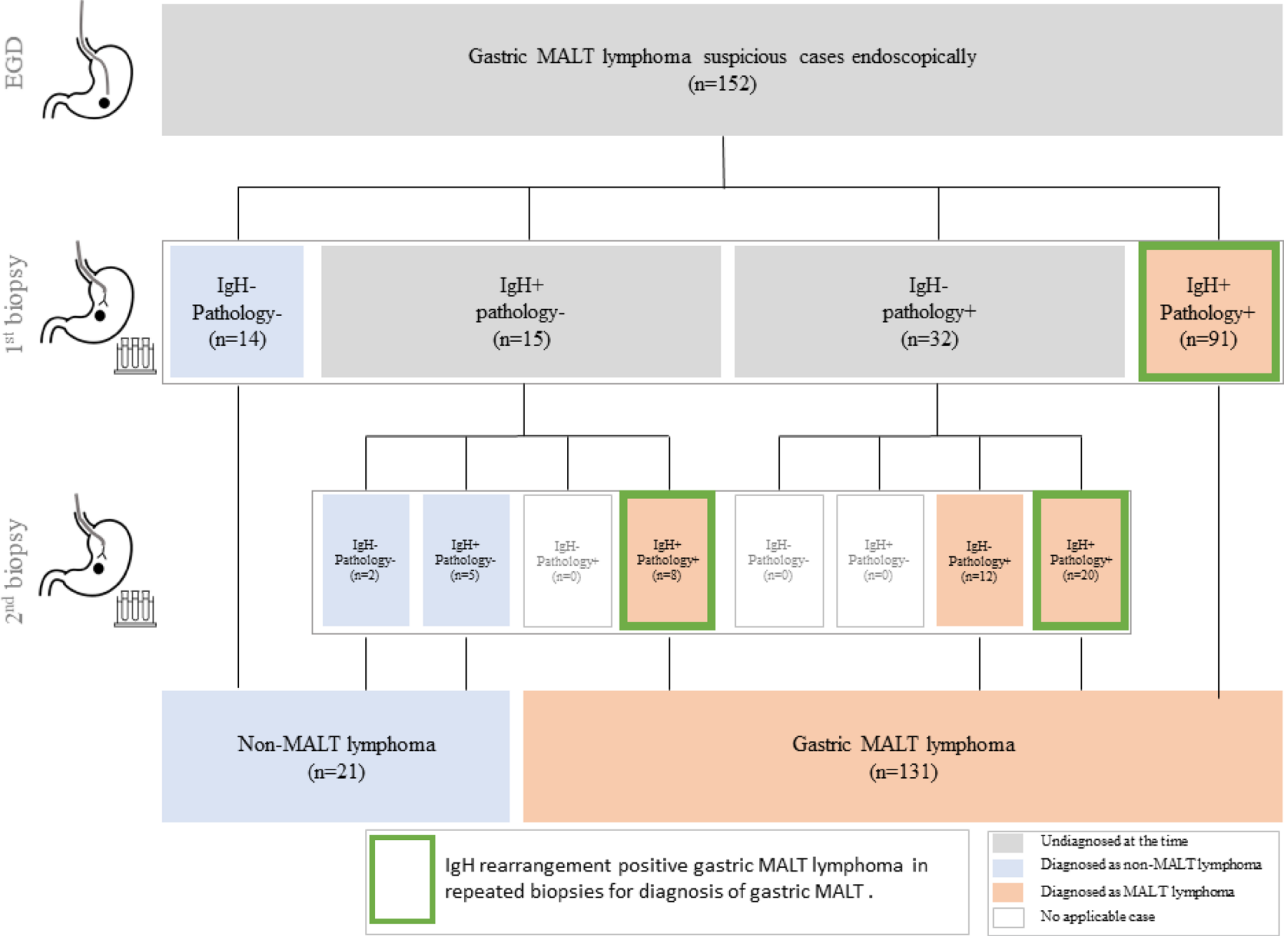
This figure outlines the inclusion criteria and patient selection flow of this study. Patients whose initial biopsy results were positive for both IgHr and pathological examination were included, whereas those who were negative for both were excluded. In cases with discrepancies between IgHr and pathological findings, a re-biopsy was performed during the second EGD. Finally, cases were included based on pathology results, which were designated as the gold standard. Notably, among the 32 cases that were initially pathological positive but were IgHr-negative, 20 (62.5%) converted to IgHr-positive upon re-biopsy; however, 12 (37.5%) remained IgHr-negative but were still included. Furthermore, of the 15 cases that were initially pathological negative but IgHr-positive, eight (53.3%) were pathological positive upon re-biopsy and were included in the study. In total, 131 cases of gastric MALT lymphoma were included in this study. The IgH rearrangement positivity rate in the first biopsy was 91/131 (69.5%). Combining first and repeat biopsies, IgHr positivity was observed in a total of 119 cases (91 + 8 + 20) (90.8%), highlighted by the green box. *MALT, mucosa-associated lymphoid tissue; EGD, esophagogastroduodenoscopy.

Defining the pathological findings with immunohistochemistry as the gold standard for diagnosis of MALT lymphoma, cases diagnosed as MALT lymphoma pathologically in either the initial biopsy or, for repeatedly biopsied cases, in the second biopsy, were defined as gastric MALT lymphoma in this study. Ultimately, 131 patients were finally confirmed to have gastric MALT lymphoma (Fig. 1). To evaluate the positivity rate of the IgHr in gastric MALT lymphoma and the usefulness of repeat biopsy, we analyzed the IgHr positivity rate in the first and second biopsies. Of the 131 cases of gastric MALT lymphoma, the proportion in which IgHr was positive in either the first or second biopsy was defined as the IgHr positive rate for gastric MALT lymphoma, and improvement of IgHr positive rate was evaluated by combining the repeated biopsies. This methodological approach was intentionally chosen to address the well-known challenge of sampling error in this disease. Gastric MALT lymphoma often presents with a heterogeneous and patchy distribution of lymphomatous infiltrates, meaning a single biopsy may not capture sufficient tumor-derived DNA, potentially leading to false-negative IgHr results. By defining positivity based on at least one positive result from two sequential biopsies, we aimed to mitigate this limitation and more accurately estimate the true positivity rate of IgHr in pathologically confirmed cases. Furthermore, we investigated the relationship between the clinicopathological features of IgHr-positive and IgHr-negative gastric MALT lymphoma.

Comprehensive evaluations with CT or PET-CT and colonoscopy were conducted for disease staging. All cases were classified as Stage I based on Lugano’s classification. After eradication therapy, EGD was performed at the 3-month follow-up, followed by surveillance every 6 months.

For patients treated after April 2018, IgHr and histopathological examinations were also performed during the follow-up period after pathological CR. Forty-one of these patients underwent histopathological examination and IgHr at the time of CR and 6 months, 1 year, and 2 years post-CR. These cases were evaluated to assess changes in the pathological findings and IgHr status during post-CR surveillance.

### Criteria for pathological diagnosis

Histopathological diagnosis, based on the identification of characteristic morphological and immunophenotypic features, was defined as the gold standard for confirming gastric MALT lymphoma in this study. We acknowledge the limitations of this approach, particularly that molecular evidence of clonality can precede overt histological features, and that sampling error can reduce diagnostic sensitivity. Our decision to use histopathology as the primary standard for inclusion was based on current clinical guidelines^12^, but our study design, which mandated re-biopsy for discrepant cases, was intended to mitigate these very issues. While a positive pathology result was used to confirm a case, a negative pathology result was not used to definitively exclude a diagnosis, especially in the presence of positive molecular findings. While molecular markers such as the BIRC3-MALT1 fusion gene or IgH gene rearrangement offer objectivity, their use as a sole gold standard is limited. The BIRC3-MALT1 fusion gene is absent in the majority (28 ~ 72%) of cases, and IgHr testing is subject to both false-negative and false-positive results^13–15^. Therefore, our approach aligns with current international guidelines^16–18^, which recommend that a definitive diagnosis be based on histopathological features, such as the presence of centrocyte-like cells and lymphoepithelial lesions (LELs), supported by immunohistochemistry. Diagnoses were primarily made according to the WHO Classification, 5th edition^19^. Particular emphasis was placed on the presence of lymphoepithelial lesions (LELs). In cases where the presence of LELs was unclear, immunostaining with CK AE1/AE3 and CD20 was used to improve diagnostic accuracy. Pathological CR was evaluated according to the GELA (Groupe d’Etude des Lymphomes de l’Adult) grading system^20^.

### Evaluation of IgHr, BIRC3-MALT1 fusion gene, and *H. pylori* infection status

**Fig. 2:**
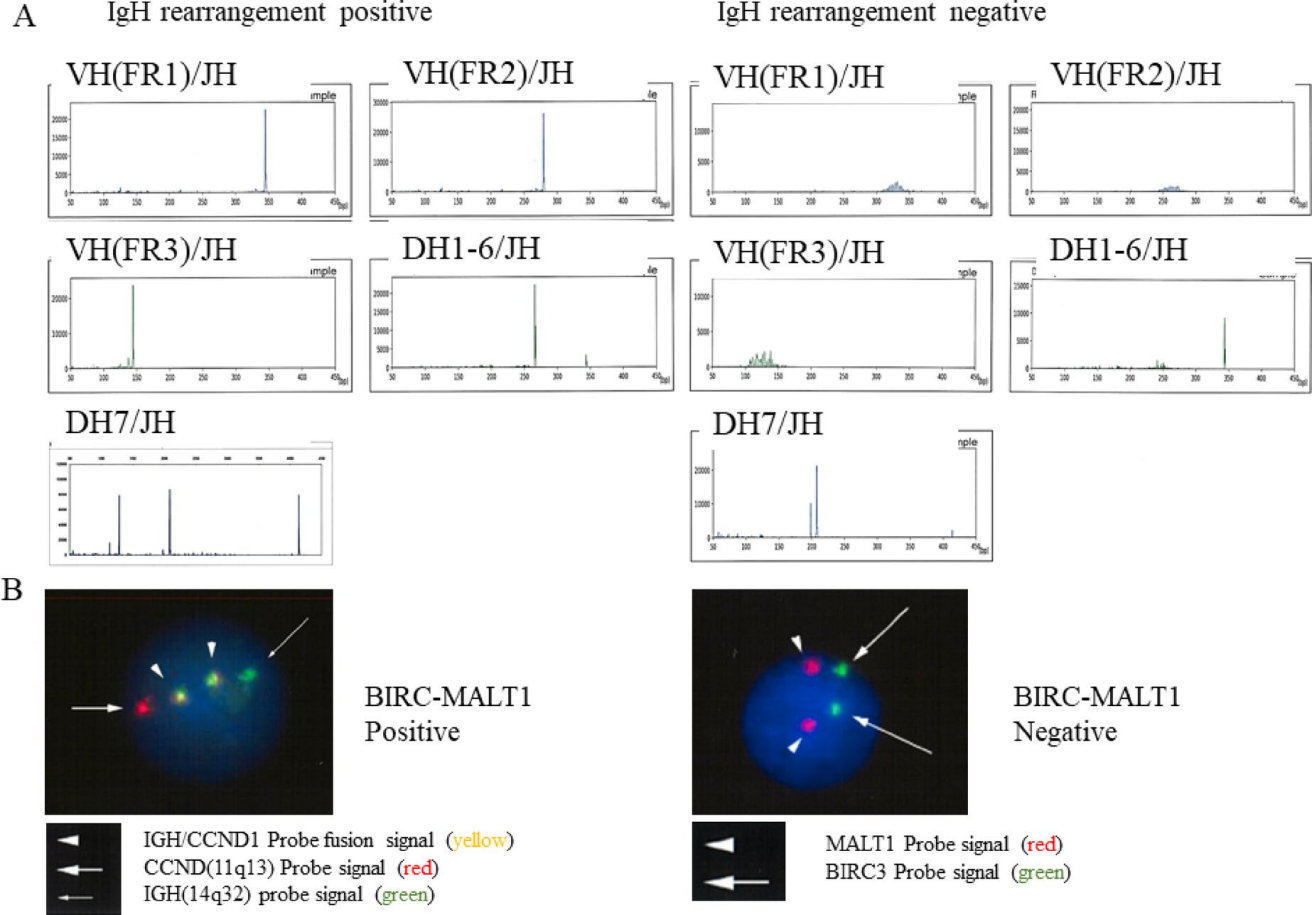
(**A**) Representative images of IgH rearrangement testing for each lesion given by the LSI Medience. (**B**) Representative images of BIRC3-MALT1-positive and BIRC3-MALT1-negative samples given by the LSI Medience.

IgHr status was assessed using a multiplex PCR assay (LSI Medience, Japan) as previously described^21^ with representative images shown in Fig. 2A. BIRC3-MALT1 chimeric transcript detection was performed by fluorescence in situ hybridization on fresh biopsy samples (LSI Medience)^22,23^, with representative images in Fig. 2B.

HP infection status was evaluated using antibody testing and endoscopic mucosal atrophy findings, classifying patients as infected, previously infected, or uninfected. Eradication success was confirmed by a urea breath test.

Anti-HP antibody levels were measured using enzyme-linked immunosorbent assays (E-plate; Eiken Chemical, Japan) from 2013 to 2021 and a latex agglutination immunoturbidimetry kit (Denka, Japan) from 2021 to 2023. The cut-off for HP-positive infection was set as 10 U/mL^24,25^. Borderline-negative cases (3–10 U/mL) were evaluated by combining antibody levels and endoscopic findings.

### Treatment strategy

Regardless of the HP infection or BIRC3-MALT1 fusion gene status, patients initially received first-line eradication therapy using a regimen that included proton pump inhibitors/Potassium competitive acid blockers (PPI/P-CAB), amoxicillin, and clarithromycin as initial treatment. In cases of eradication failure in patients who were HP-positive, second-line eradication therapy with a PPI/P-CAB, amoxicillin, or metronidazole was administered. Notably, patients with gastric MALT lymphoma who did not achieve CR with eradication therapy subsequently underwent radiation therapy (RT) or chemotherapy.

### Statistical analysis

Continuous variables are presented as mean ± standard deviation and categorical variables as numbers and percentages. Between-group differences were evaluated using the Mann–Whitney U test or Student’s t-test for quantitative data and the χ2 test or Fisher’s exact test for categorical data, appropriately. Statistical significance was set at two-sided P values < 0.05, and all analyses were performed using the EZR software version 1.68 (Saitama Medical Center, Jichi Medical University, Saitama, Japan)^26^.

## Results

### Pathology and IgHr status results in diagnostic biopsies

As shown in Fig. 1, 14 of the 152 patients with suspected MALT lymphoma who were IgHr- and pathology-negative on the first biopsy were excluded. Additionally, 91 patients who were IgHr-positive and pathology-positive at the first biopsy were included. For the 47 cases with discordant results between IgHr and pathological findings (15 IgHr-positive/pathology-negative and 32 cases IgHr-negative/pathology-positive) at the first biopsy, EGD with biopsy was repeated for further evaluation. Among the 32 cases that were initially pathological (+) but IgHr (-), 20 cases (62.5%) converted to IgHr (+) upon re-biopsy; however, 12 cases (37.5%) remained IgHr (-). As pathological findings were set as the gold standard, these 32 cases were diagnosed with gastric MALT lymphoma. Furthermore, eight (53.3%) of the 15 cases that were initially pathological (-) but IgHr (+) converted to pathological (+) and were diagnosed with gastric MALT lymphoma upon re-biopsy. Seven cases which were pathologically negative upon re-biopsy were excluded. Notably, all seven of these IgHr-positive/pathology-negative cases were also confirmed to be negative for the BIRC3-MALT1 fusion gene. Of 152 cases, 131 were diagnosed with gastric MALT lymphoma, and 21 cases were excluded from this study.

### Patient backgrounds

Patient backgrounds and treatment outcomes are summarized in Table 1. Among the 131 patients with histologically confirmed gastric MALT lymphoma, the mean age was 63.6 years, with 60 males (46%). Active HP infection was observed in 54 patients (41.2%), a previous infection in 25 (19.1%), and no infection in 52 (39.7%). Furthermore, atrophy of the background gastric mucosa was assessed following the Kimura–Takemoto classification^27^: non, closed-, and open-type atrophy accounted for 52 (39.7%), 39 (29.8%), and 40 (30.5%) cases, respectively. The tumor locations included the lower (L), middle (M), and upper (U) regions of the stomach, with the following distributions: 21 cases in the L region (16%), 68 in M (52.3%), 25 in U (19.2%), seven in M + L (5.4%), three in U + M (2.3%), one in U + L (0.8%), and six involving all three regions (U + M + L, 4.6%). Solitary lesions were observed in 55 patients (42.0%), whereas multiple lesions were found in 76 (58.0%), with several cases spanning multiple gastric regions.

**Table 1:** Background information of localized gastric MALT lymphoma analyzed in this study.

		Gastric MALT lymphoma (*n* = 131)
Average age, years ± SD		63.6 ± 11.42
Sex	Male	60 (46)
	Female	71 (54)
Hp infection	Current	54 (41.2)
	Previous	25 (19.1)
	Uninfected	52 (39.7)
Mucosal atrophy	None	52 (39.7)
	Closed type	39 (29.8)
	Open type	40 (30.5)
Locations in the stomach	L	21 (16.0)
	M	68 (52.3)
	U	25 (19.2)
	M/L	7 (5.4)
	U/M	3 (2.3)
	U/L	1 (0.8)
	U/M/L	6 (4.6)
Number of lesions	Single	55 (42.0)
	Multiple	76 (58.0)
Morphological type	Superficial	103 (78.6)
	Submucosal tumor	12 (9.2)
	Nodular gastritis-like	6 (4.6)
	Ulceration	5 (3.8)
	Others	5 (3.8)
BIRC3-MALT1 fusion gene	Positive	36 (28.1)
	Negative	95 (72.5)
Efficacy of eradication therapy	CR	63 (49.6)
	NC	64 (50.4)
Treatment	Eradication only	68 (51.9)
	Eradication + radiation therapy	51 (38.9)
	Eradication + chemotherapy	8 (6.1)
	Others	4 (3.1)

Numbers in parentheses represent percentages.

*Including overlaps.

HP, *Helicobacter pylori*; CR, complete response; NC, no change; U, upper part of the stomach; M, middle part of the stomach; L, lower part of the stomach, MALT, mucosa-associated lymphoid tissue; SD, standard deviation.

Macroscopic classification based on endoscopic morphology showed that superficial-type lesions were the most common (103 cases, 78.6%), followed by submucosal tumor-like lesions (SMT) in 12 cases (9.2%), nodular gastritis-like morphology in six cases (4.6%), and ulcerative lesions in five cases (3.8%). The BIRC3-MALT1 fusion gene was positive in 36 cases (28.1%).

All patients received HP eradication therapy, and CR was achieved in 63 (49.6%) patients following primary and secondary eradication therapies. Among the patients who were unresponsive to eradication therapy, additional RT was conducted in 51 cases (38.9%) and chemotherapy in eight cases (6.1%).

### Comparison of IgHr positive rates between single and repeat biopsies

As shown in Fig. 1, among the 131 cases ultimately diagnosed as gastric MALT lymphoma, 91 were positive for IgHr in the initial biopsy. This corresponds to an IgHr positivity rate of 69.5% (91/131). In cases where pathological findings and IgHr results from the first biopsy were discrepant, a second biopsy was performed.

**Fig. 3:**
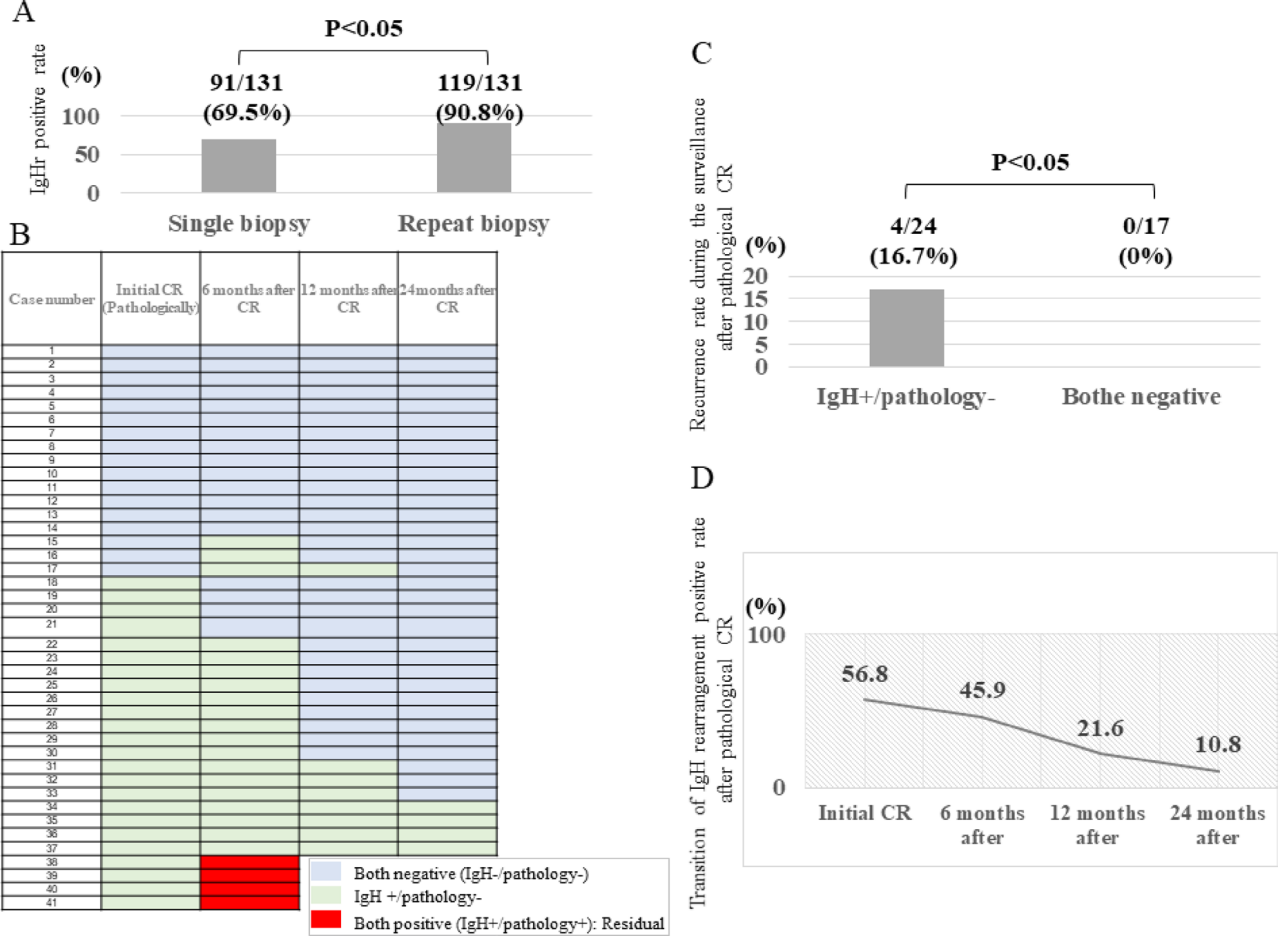
(**A**) Comparison of IgHr positive rate between single biopsy and repeat biopsy. IgHr in single and repeat biopsies was 69.5% and 90.8%, respectively. IgHr positive rate increased significantly by repeat biopsy. (**B**) IgHr and pathological status post-pathological complete response (CR). Among the 17 patients who were IgHr-negative and pathological negative upon achieving an initial pathological CR, all remained negative after 2 years. Of the 24 pathological negative but IgHr-positive cases at the time of pathological CR, 16 (66.7%) converted to both negative, four (16.7%) remained IgHr-positive after 2 years, and four (16.7%) experienced recurrence based on pathological findings. (**C**) Recurrence rate based on initial IgHr status at pathological CR. A comparison between cases with IgHr-positivity on pathological CR and those with both IgHr-negative and pathological negative statuses revealed a recurrence rate of 16.7% in IgHr-positive cases, indicating a significantly higher recurrence frequency in this group. (**D**) IgHr positivity trends following pathological CR. The rate of IgHr positivity in patients who achieved pathological CR gradually decreased over time, suggesting a potential time lag between achieving pathological CR and the subsequent IgHr-negative conversion.

When combining the results of both the first and second biopsies, IgHr was detected in a total of 119 cases (91 + 8 + 20), as highlighted by the green box in Fig. 1. This yielded an overall IgHr positivity rate of 90.8% (119/131). The inclusion of repeat biopsies significantly increased the detection rate of IgHr compared to a single biopsy (Fig. 3A).

### Concordance between diagnostic modalities

To assess the relationship between the different diagnostic methods used, we analyzed the concordance rates among pathological diagnosis, IgHr positivity, and BIRC3-MALT1 fusion gene status in the final cohort of 131 gastric MALT lymphoma cases (Table 2). The final concordance rate between pathological diagnosis and IgHr positivity was 90.8% (119/131). As expected, the concordance with BIRC3-MALT1 positivity was lower, at 27.5% (36/131) for pathological diagnosis and 29.4% (35/119) for IgHr positivity, reflecting the known prevalence of this specific fusion gene. Among the 36 cases with a positive BIRC3-MALT1 fusion gene, 35 (97.2%) were also IgHr-positive.

**Table 2:** Cross-tabulation of diagnostic modalities in the gastric MALT lymphoma cohort (*n* = 131).

	Pathologically diagnosed MALT lymphoma	IgHr positive	BIRC3-MALT1fusion gene positive
Pathologically diagnosed MALT lymphoma (*n* = 131)		119 (90.8)	36 (27.5)
IgHr positive (*n* = 119)	119 (100)		35 (29.4)
BIRC3-MALT1fusion gene positive (*n* = 36)	36 (100)	35 (97.2)	

Concordance rate; %.

IgHr, Immunoglobulin Heavy chain gene rearrangement; MALT, Mucosa-Associated Lymphoid Tissue.

### Comparison of clinicopathological characteristics between IgHr-positive and negative cases

Table 3 presents a comparison of the clinicopathological characteristics between the IgHr-positive and negative groups. The groups showed no significant differences regarding sex, tumor location in the stomach, number of lesions, morphological type, or existence of the BIRC3-MALT1 fusion gene. However, the IgHr-positive group had a significantly lower mean age (56.1 ± 11.5 vs. 67.8 ± 8.4 years; *P* = 0.037), a greater proportion of active *HP* infections (75.0% vs. 37.8%; *P* = 0.027), and a higher prevalence of mucosal atrophy (91.7% vs. 57.1%; *P* = 0.027) than the IgHr-negative group. Furthermore, eradication therapy was significantly more effective in the IgHr-positive group (83.3% vs. 38.7%; *P* = 0.013) than in the negative group.

**Table 3:** Comparison of clinicopathological features between IgHr-positive and -negative cases.

		IgHr positive *n* = 119	IgHr negative *n* = 12	*P*-value
Average age, years ± SD		67.8 ± 8.4	56.1 ± 11.5	0.037*
Sex	Male	62 (52.1)	9 (75.0)	0.223
	Female	57 (47.9)	3 (25.0)	
Hp infection	Negative (Naïve/previous)	74 (62.2)	3 (25.0)	0.027*
	Positive (Current)	45 (37.8)	9 (75.0)	
Mucosal atrophy	Absent	51 (42.9)	1 (8.3)	0.027*
	Present	68 (57.1)	11 (91.7)	
Location in the stomach (Including overlaps)	L/M	108 (90.7)	10 (83.3)	1.000
	U	32 (26.9)	3 (25.0)	
Number of lesions	Single	51 (42.9)	4 (33.3)	0.760
	Multiple	68 (57.1)	8 (66.7)	
Morphological type	Superficial	94 (79.0)	9 (75.0)	0.719
	Others	25 (21.0)	3 (25.0)	
	Submucosal tumor	11 (9.2)	1 (8.3)	
	Nodular-gastritis-like	5 (4.2)	1 (8.3)	
	Ulceration	4 (3.4)	1 (8.3)	
	MLP	5 (4.2)	0 (0)	
BIRC3-MALT1 fusion gene	Positive	35 (29.4)	1 (8.3)	0.178
	Negative	84 (70.6)	11 (91.7)	
Efficacy of eradication therapy	CR	46 (38.7)	10 (83.3)	0.013*
	NC	59 (61.3)	2 (16.7)	

Numbers in parentheses represent percentages.

*Statistically significant.

HP, *Helicobacter pylori*; IgHr, immunoglobulin H rearrangement test; CR, complete response; NC, no change; U, upper stomach; M, middle stomach; L, lower stomach; MLP, multiple lymphomatous polyposis; MALT, mucosa-associated lymphoid tissue.

### Changes in IgHr status and pathological findings in pathological CR cases in a longitudinal follow-up for two years after pathological CR

A longitudinal follow-up was conducted on IgHr status and pathological findings (Fig. 3B) for the 41 patients who achieved pathological CR and were monitored for over 2 years using IgHr and pathological assessments. Of the 17 cases that were negative for pathology and IgHr at the time of CR (both negative cases), three cases temporarily showed IgHr positivity; however, all cases reverted to negative IgHr status with negative pathological findings by the 2-year mark, confirming CR status (Fig. 3B).

In contrast, among the 24/41 cases (58.5%) that were IgHr positive but pathological (-) at the time of CR (IgHr+/Pathology- cases), 4/24 (16.7%) cases showed pathological recurrence, a significantly higher recurrence rate than the 0/17 (0%) cases observed in the initially both-negative group (Fig. 3B and C).

Further analysis of the 37 cases, excluding the four recurrent cases, revealed that 21/37 cases (56.8%) were IgHr-positive at the time of CR. However, IgHr positivity decreased progressively over time: at 6 months, 17/37 cases (45.9%); at 1 year, 8/37 cases (21.6%); and at 2 years, 4/37 cases (10.8%) (Fig. 3B and D). This trend suggests a possible time lag between achieving pathological CR and conversion to IgHr-negativity.

## Discussion

In this study, we assessed the clinical significance of IgHr in managing gastric MALT lymphoma. Integrating the results of two sequential biopsies improved the IgHr-positivity rate. IgHr-positive cases had higher H. pylori infection rates and better CR rates after eradication than IgHr-negative cases. Patients with IgHr positivity at pathological CR had a higher recurrence rate compared to IgHr negative cases. IgHr positivity declined over time, indicating a time lag between pathological CR and IgH-negative conversion.

A key finding of this study was the initial IgHr positivity rate of 69.5% in pathologically confirmed MALT lymphoma cases. While this may seem low, it is crucial to interpret this figure within the context of the inherent diagnostic challenges of this disease. This rate is, in fact, comparable to or even higher than those reported in previous studies, which range from 48.4 to 50%^9,16^. This initial concordance rate likely does not invalidate the diagnoses; rather, it may be attributed to a combination of factors, including the possibility of sampling error stemming from the patchy, multifocal nature of gastric MALT lymphoma. The subsequent significant increase in the positivity rate to 90.8% upon repeat biopsy may support this interpretation. This improvement demonstrates the efficacy of repeated sampling in overcoming initial diagnostic limitations and enhancing the overall diagnostic yield, rather than indicating a flaw in the initial cohort selection.

Reports on IgHr positivity rates in MALT lymphomas are mostly limited to small case series. Relatively larger studies among previous reports documented IgHr positivity in 50 of 86 cases (58.1%) of thyroid MALT lymphoma^28^ and in 37 of 60 cases (61.7%) of MALT lymphomas of mixed origins, including the stomach, salivary glands, intestines, and orbit^14^. For gastrointestinal MALT lymphoma, a 2014 study of 50 patients reported IgHr positivity in 48.4% of stage I–II and 90% of stage III–IV MALT cases^13^. Specifically for gastric MALT lymphomas, a smaller series reported IgHr positivity in six of 12 cases (50%)^16^. Additionally, a study reported a 50% positivity in six cases, 30 specimens with low-grade MALT lymphoma; 60% positivity in 13 cases, 45 specimens with high-grade MALT lymphoma; and 80.6% in nine cases, 36 specimens with diffuse large B-cell lymphoma^29^. In this study, we analyzed a relatively large cohort and observed IgHr positivity in 91 of 131 cases (69.5%) in the initial biopsy, a rate consistent with previous reports. Notably, repeat biopsy increased the IgHr positivity rate to 119 of 131 cases (90.8%), which tends to be higher than previously reported rates. This significant improvement in the detection rate warrants consideration of potential bias, such as that arising from “some kind of assumption or misleading diagnosis.” However, we argue that the observed increase is a genuine finding rather than a procedural artifact. The multiplex PCR analysis for IgHr is an objective molecular test that yields a binary positive/negative result, making it less susceptible to the kind of interpretive bias that can affect histopathological diagnosis. In our study, these analyses were performed by an external laboratory whose technicians were blinded to all clinical information, including previous biopsy results. This procedural objectivity supports our conclusion that the increased IgHr positivity is attributable to obtaining a more representative tissue sample during the second biopsy—thereby overcoming initial sampling error—and not to any assumption-driven or misleading diagnostic bias.

The clinical utility of IgHr testing, particularly in relation to fusion gene analysis, warrants detailed discussion. From a genetic standpoint, it is reasonable to question the necessity of IgHr testing in cases positive for the BIRC3-MALT1 fusion gene. Since the t(11;18)(q21;q21) translocation occurs in B-cells that have already completed V(D)J recombination, a BIRC3-MALT1–positive case should theoretically also be IgHr-positive^30–32^. Our findings strongly support this, as 97.2% (35/36) of BIRC3-MALT1–positive cases in our cohort were indeed IgHr-positive. However, the core rationale for performing IgHr testing more broadly is that the BIRC3-MALT1 fusion gene is present in only a minority of gastric MALT lymphomas, with reported rates of approximately 24–32%^33–35^. Consequently, for the majority of cases that are BIRC3-MALT1–negative, the detection of B-cell clonal proliferation via IgHr analysis remains an indispensable diagnostic tool. Our study’s primary objective was therefore to evaluate the diagnostic performance of a modern multiplex PCR-based assay for IgHr in a real-world clinical cohort, which offers superior accuracy to less reliable methods like immunohistochemistry^17,36^. This evaluation was essential to clarify the role of IgHr testing for the large subset of patients in whom specific fusion gene analysis is not informative.

Repeat biopsy also contributed to pathological diagnosis. Among the 106 cases initially positive for IgHr, 15 were not diagnosed as gastric MALT lymphoma during the first biopsy. However, eight of these cases (53.3%) were histopathologically confirmed as MALT lymphoma upon re-biopsy. Similar scenarios are described in case reports in the literature. In one report, a patient with conjunctival MALT lymphoma initially showed IgHr positivity without pathological evidence of MALT lymphoma^11^. This prompted ongoing monitoring, which eventually led to a definitive histopathological diagnosis of MALT lymphoma 11 months later by repeated biopsy. The other case involved bronchus-associated lymphoid tissue lymphoma^37^. Here, despite the absence of pathological evidence of MALT lymphoma, IgHr positivity supported the presumed diagnosis of MALT lymphoma, which was followed by successful chemotherapy. There are currently no large-scale studies detailing the outcomes of patients with histopathological negative yet IgHr-positive findings. Therefore, whether chemotherapy should be initiated solely based on IgHr positivity remains debatable. However, our findings suggest that in cases of discrepancy between the IgHr-based diagnosis and histopathological findings during the initial examination, performing a re-biopsy may lead to a more appropriate diagnosis of MALT lymphoma. It is particularly important to consider the possibility of a positive conversion in pathology in cases with IgHr positivity but negative pathological findings which may later lead to the diagnosis of MALT lymphoma during follow-up.

To improve the clinical applicability of these findings, our study offers further guidance on differentiating true MALT lymphoma from the potential false positives seen in reactive B-cell clonal proliferation, such as in H. pylori gastritis. In cases where IgHr is positive but definitive MALT lymphoma is not identified, a meticulous correlation with histopathology is crucial. This includes performing a thorough pathological examination, aided by ancillary immunohistochemistry (e.g., CD20, AE1/AE3), to clarify the nature of B-cell infiltration and to confirm the absence of characteristic features like LELs or disrupted follicular polarity^9,18^. Furthermore, as demonstrated in our study, when initial findings are discordant, performing repeat biopsies for both pathological and molecular assessment is a valuable diagnostic strategy. This approach not only helps to overcome initial sampling error but also allows for temporal monitoring. Some reports suggest that the consistent detection of the same B-cell clone over time is more indicative of a neoplastic process like MALT lymphoma than a transient reactive one^38,39^. Therefore, an integrated approach that combines careful pathology with repeat testing is essential for accurate differentiation and management.

Regarding the relationship between IgHr status and HP infection or efficacy of eradication therapy, our study indicates that IgHr positivity is higher in HP-positive gastric MALT lymphomas, and patients who were IgHr positive responded significantly better to eradication therapy than those who were not. However, a detailed prognostic assessment was not conducted. Previous research has shown a relationship between IgHr positivity and prognosis; for example, in orbital MALT lymphomas, IgHr-positive cases tend to have higher recurrence rates than negative cases^40^. Moreover, in gastric MALT lymphoma, IgHr positivity correlates with the disease stage, and patients with IgHr positivity demonstrate significantly shorter overall survival^13^. Therefore, despite the favorable response to eradication in IgHr-positive cases, these previous reports suggest a potentially poor prognosis, underscoring the need for further investigation. The high CR rate achieved by eradication therapy in IgHr-positive cases may, in part, be attributable to the higher prevalence of Helicobacter pylori (HP) infection in this subgroup.

Furthermore, only a few reports have addressed the IgHr status after CR. In one study on 12 gastric MALT lymphoma cases, six were IgHr-positive; of those, one (16.7%) remained IgHr-positive post-CR^16^. This finding highlights the possibility that IgHr positivity may persist in some cases even after pathological CR, although these observations stem from small sample sizes. Our findings provide valuable insights through the tracking of IgHr trends over 2 years post-CR in 41 patients. Notably, IgHr positivity declined from a positive rate of 56.8% at initial pathological CR to a positive rate of 10.8% at 2 years post-CR, indicating a potential delay between pathological CR and IgHr-negative conversion.

Notably, among the patients with pathological CR and initial IgHr positivity, 16.7% showed histopathological evidence of residual or recurrent MALT lymphoma. Therefore, in cases where IgHr remains positive after CR, it is essential to consider the potential delay in IgHr clearance and the risk of residual disease relapse. These findings underscore the need for a vigilant follow-up. Surveillance for gastric MALT lymphoma may benefit from evaluating pathological findings and IgHr status to predict disease relapse more effectively.

Our study highlights the complex interplay and occasional discordance between histopathology, IgHr testing, and fusion gene analysis, which is crucial for accurate diagnosis. Several types of discordance were observed, each with distinct underlying reasons.

First, the discordance between pathological diagnosis and BIRC3-MALT1 status (27.5% concordance) is an expected finding, as the BIRC3-MALT1 fusion gene is a specific but not universal feature of gastric MALT lymphoma, known to be present in only a subset of cases^33–35^. Therefore, its absence does not rule out the diagnosis.

Second, the discordance between pathological diagnosis and IgHr positivity (90.8% concordance) is central to our investigation. The 12 cases that remained IgHr-negative despite a definitive pathological diagnosis exemplify the issue of false-negative IgHr results. These can be attributed to several factors, including sampling error from the patchy nature of the disease or technical limitations, such as PCR primers that do not cover the full spectrum of possible V(D)J recombination patterns. The question of whether these pathologically positive but IgHr-negative cases truly represent MALT lymphoma is complex. However, given the well-documented limitations of IgHr testing sensitivity, our study sided with the definitive pathological findings as the basis for diagnosis.

Finally, the near-perfect concordance between BIRC3-MALT1 positivity and IgHr positivity (97.2%) aligns with biological expectations, with the single discordant case likely representing a sampling or technical artifact. Collectively, these findings demonstrate that while each test provides valuable information, no single modality is infallible. They reinforce the necessity of an integrated diagnostic approach that critically evaluates the results from all methods to reach the most accurate conclusion.

This study has some limitations. First, this study was a single-center retrospective investigation, and while the number of cases included was relatively high compared with previous reports^13,16^, the rarity of this condition means that the sample size may still be considered insufficient for drawing definitive conclusions. Furthermore, the retrospective nature of the study introduces the potential for diagnostic bias; for instance, a pathologist’s awareness that a biopsy was a repeat sample could have subtly influenced their interpretation, although we sought to mitigate this by adhering to strict WHO criteria and having diagnoses performed by pathologists specializing in gastrointestinal lymphoma.

Second, our use of histopathological diagnosis as the gold standard, while consistent with current clinical practice, has inherent limitations. This approach may overlook biologically indolent but molecularly clonal lesions that lack definitive histological features. Our study design sought to mitigate this issue through the mandatory re-evaluation of all discrepant cases, which successfully led to the inclusion of several cases that converted to pathology-positive upon re-biopsy. However, a key limitation is the exclusion of cases that remained pathologically negative despite positive IgHr findings. To maintain the rigor of our analysis within a strictly defined, pathologically confirmed cohort, these patients were excluded. We fully acknowledge the clinical significance of this group—consisting of patients who were IgHr-positive, BIRC3-MALT1 fusion gene-negative, but lacked pathological confirmation even after repeat biopsies. Their exclusion does not imply an absence of disease; on the contrary, it is conceivable that some of these cases represent true MALT lymphoma with very early or subtle lesions. This highlights a population that requires vigilant, long-term surveillance, where repeated biopsy remains an essential diagnostic tool.

A third major limitation is the inability to perform a robust multivariate analysis to identify independent predictors of recurrence. Such an analysis, using logistic regression or Cox proportional hazards modeling, would be necessary to adjust for potential confounders and strengthen our conclusions regarding recurrence risk. However, our follow-up cohort included only four recurrence events. This extremely low number of events makes it statistically unfeasible to build a stable multivariate model. The low Events Per Variable (EPV) ratio leads to statistical issues such as quasi-complete separation, which prevents model convergence and produces unreliable estimates. Therefore, while our study suggests an association between IgHr status and recurrence, this finding is based on simple comparative statistics and should be considered preliminary. Larger prospective studies are required to accumulate a sufficient number of events to perform a robust multivariate analysis and validate these initial findings.

## Conclusion

In this study, we evaluated the utility of IgHr for the diagnosis, treatment response assessment, and surveillance of gastric MALT lymphoma. Our findings highlight the importance of repeat biopsy for accurate diagnosis and suggest that even when histopathological findings are negative, IgHr positivity—indicating monoclonal immunoglobulin proliferation—may signify a risk of histopathological progression to MALT lymphoma or residual disease, necessitating careful follow-up. Additionally, a time lag may exist between pathological complete remission and IgHr negativity. This potential delay should be considered when interpreting surveillance results.

## Data Availability

Data from this study are available upon request from the corresponding author.

## References

[1] Takigawa, H. et al. Involvement of non-Helicobacter pylori Helicobacter infections in Helicobacter pylori-negative gastric MALT lymphoma pathogenesis and efficacy of eradication therapy. *Gastric Cancer* **24**(4), 937–945 (2021). 33638751 10.1007/s10120-021-01172-x

[2] Takigawa, H. et al. Helicobacter suis infection is associated with nodular gastritis-like appearance of gastric mucosa-associated lymphoid tissue lymphoma. *Cancer Med.* **8**(9), 4370–4379 (2019). 31210418 10.1002/cam4.2314 PMC6675707

[3] Isaacson, P. & Wright, D. H. Extranodal malignant lymphoma arising from mucosa-associated lymphoid tissue. *Cancer* **53**(11), 2515–2524 (1984). 6424928 10.1002/1097-0142(19840601)53:11<2515::aid-cncr2820531125>3.0.co;2-c

[4] Papadaki, L., Wotherspoon, A. C. & Isaacson, P. G. The lymphoepithelial lesion of gastric low-grade B-cell lymphoma of mucosa-associated lymphoid tissue (MALT): An ultrastructural study. *Histopathology* **21**(5), 415–421 (1992). 1452124 10.1111/j.1365-2559.1992.tb00425.x

[5] El-Zimaity, H. M. et al. The differential diagnosis of early gastric mucosa-associated lymphoma: Polymerase chain reaction and paraffin section immunophenotyping. *Mod. Pathol.* **12**(9), 885–893 (1999). 10496597

[6] Cho, J. Basic immunohistochemistry for lymphoma diagnosis. *Blood Res.* **57**(S1), 55–61 (2022). 35483927 10.5045/br.2022.2022037 PMC9057666

[7] Aiello, A. et al. PCR-based clonality analysis: a reliable method for the diagnosis and follow-up monitoring of conservatively treated gastric B-cell MALT lymphomas? *Histopathology* **34**(4), 326–330 (1999). 10231400 10.1046/j.1365-2559.1999.00628.x

[8] Yi, Z. H. et al. Combined histology and molecular biology for diagnosis of early stage gastric MALT lymphoma. *Chin. J. Dig. Dis.* **7**(1), 12–18 (2006). 16412032 10.1111/j.1443-9573.2006.00238.x

[9] Hsi, E. D. et al. Detection of Immunoglobulin heavy chain gene rearrangement by polymerase chain reaction in chronic active gastritis associated with *Helicobacter pylori*. *Hum. Pathol.* **27**(3), 290–296 (1996). 8600045 10.1016/s0046-8177(96)90071-4

[10] Montalban, C. et al. Regression of gastric high grade mucosa associated lymphoid tissue (MALT) lymphoma after *Helicobacter pylori* eradication. *Gut* **49**(4), 584–587 (2001). 11559658 10.1136/gut.49.4.584 PMC1728454

[11] Fukuhara, J. et al. Conjunctival lymphoma arising from reactive lymphoid hyperplasia. *World J. Surg. Oncol.* **10**, 194 (2012). 22985187 10.1186/1477-7819-10-194 PMC3499207

[12] Alaggio, R. et al. The 5th edition of the World Health Organization classification of haematolymphoid tumours: Lymphoid neoplasms. *Leukemia* **36**(7), 1720–1748 (2022). 35732829 10.1038/s41375-022-01620-2 PMC9214472

[13] Zhang, G. P., Cao, P. F. & Feng, L. J. Detection and clinical significance of genes in primary gastrointestinal MALT lymphoma. *Tumour Biol.* **35**(4), 3223–3228 (2014). 24272086 10.1007/s13277-013-1421-8

[14] Zhang, J. F., Zhang, S. X. & Li, J. Molecular pathological diagnosis of mucosa-associated lymphoid tissue lymphoma. *Zhongguo Shi Yan Xue Ye Xue Za Zhi* **23**(3), 689–692 (2015). 10.7534/j.issn.1009-2137.2015.03.017 26117018

[15] Takano, Y. et al. Clonal Ig-gene rearrangement in some cases of gastric RLH detected by PCR method. *Pathol. Res. Pract.* **188**(8), 973–980 (1992). 1300609 10.1016/S0344-0338(11)81240-9

[16] Akahane, M. et al. Detection of monoclonality of gastric MALT lymphoma using PCR method and its clinicopathological application. *Rinsho byori Jpn. J. Clin. Pathol.* **51**(9), 852–858 (2003). 14560652

[17] Burke, J. S. Lymphoproliferative disorders of the Gastrointestinal tract: A review and pragmatic guide to diagnosis. *Arch. Pathol. Lab. Med.* **135**(10), 1283–1297 (2011). 21970484 10.5858/arpa.2011-0145-RA

[18] Hummel, M. et al. Wotherspoon criteria combined with B cell clonality analysis by advanced polymerase chain reaction technology discriminates Covert gastric marginal zone lymphoma from chronic gastritis. *Gut* **55**(6), 782–787 (2006). 16423889 10.1136/gut.2005.080523 PMC1856242

[19] Nagtegaal, I. D. et al. The 2019 WHO classification of tumours of the digestive system. *Histopathology* **76**(2), 182–188 (2020). 31433515 10.1111/his.13975 PMC7003895

[20] Copie-Bergman, C. et al. Proposal for a new histological grading system for post-treatment evaluation of gastric MALT lymphoma. *Gut* **52**(11), 1656 (2003). 14570741 10.1136/gut.52.11.1656 PMC1773845

[21] van Dongen, J. J. et al. Design and standardization of PCR primers and protocols for detection of clonal Immunoglobulin and T-cell receptor gene recombinations in suspect lymphoproliferations: report of the BIOMED-2 concerted action BMH4-CT98-3936. *Leukemia* **17**(12), 2257–2317 (2003). 14671650 10.1038/sj.leu.2403202

[22] Streubel, B. et al. T(14;18)(q32;q21) involving IGH and MALT1 is a frequent chromosomal aberration in MALT lymphoma. *Blood* **101**(6), 2335–2339 (2003). 12406890 10.1182/blood-2002-09-2963

[23] Akagi, T. et al. A novel gene, MALT1 at 18q21, is involved in t(11;18) (q21;q21) found in low-grade B-cell lymphoma of mucosa-associated lymphoid tissue. *Oncogene* **18**(42), 5785–5794 (1999). 10523859 10.1038/sj.onc.1203018

[24] Kotachi, T. et al. Serological evaluation of gastric cancer risk based on pepsinogen and *Helicobacter pylori* antibody: Relationship to endoscopic findings. *Digestion* **95**(4), 314–318 (2017). 28571035 10.1159/000477239

[25] Shuto, M. et al. Association between gastric cancer risk and serum *Helicobacter pylori* antibody titers. *Gastroenterol. Res. Pract.* **2017**, 1286198 (2017). 10.1155/2017/1286198 PMC5485312 28690637

[26] Kanda, Y. Investigation of the freely available easy-to-use software ‘EZR’ for medical statistics. *Bone Marrow Transpl.* **48**(3), 452–458 (2013). 10.1038/bmt.2012.244 PMC3590441 23208313

[27] Kimura, K. An endoscopic recognition of the atrophic border and its significance in chronic gastritis. *Endoscopy* **1**(03), 87–97 (1969).

[28] Suzuki, A. et al. Flow cytometric, gene rearrangement, and karyotypic analyses of 110 cases of primary thyroid lymphoma: A single-institutional experience in Japan. *Endocr. J.* **66**(12), 1083–1091 (2019). 31484843 10.1507/endocrj.EJ18-0348

[29] Kido, S., Miyazaki, K. & Tokunaga, O. The relationship between primary gastric B-cell lymphoma and Immunoglobulin heavy chain (IgH) gene rearrangement–a histopathological study of primary gastric lymphomas. *Pathol. Res. Pract.* **199**(10), 647–658 (2003). 14666967 10.1078/0344-0338-00476

[30] Rossi, D., Bertoni, F. & Zucca, E. Marginal-Zone lymphomas. *N. Engl. J. Med.* **386**(6), 568–581 (2022). 35139275 10.1056/NEJMra2102568

[31] Du, M. Q. MALT lymphoma: Genetic abnormalities, immunological stimulation and molecular mechanism. *Best Pract. Res. Clin. Haematol.* **30**(1–2), 13–23 (2017). 28288707 10.1016/j.beha.2016.09.002

[32] Xia, H. et al. Analysis of API2-MALT1 fusion, trisomies, and immunoglobulin VH genes in pulmonary mucosa-associated lymphoid tissue lymphoma. *Hum. Pathol.* **42**(9), 1297–1304 (2011). 21396678 10.1016/j.humpath.2010.11.022

[33] Jung, H. et al. Clinical impact assessment and utilization prospects of the t(11:18) chromosomal translocation in gastric MALT lymphoma in Koreans: A single center retrospective analysis. *Korean J. Gastroenterol.* **81**(1), 29–35 (2023). 10.4166/kjg.2022.129 PMC12285382 36695064

[34] Cascione, L. et al. Novel insights into the genetics and epigenetics of MALT lymphoma unveiled by next generation sequencing analyses. *Haematologica* **104**(12), e558–e561 (2019). 31018978 10.3324/haematol.2018.214957 PMC6959164

[35] Raderer, M., Kiesewetter, B. & Du, M. Q. Clinical relevance of molecular aspects in extranodal marginal zone lymphoma: A critical appraisal. *Ther. Adv. Med. Oncol.* **15**, 17588359231183565 (2023). 37389189 10.1177/17588359231183565 PMC10302523

[36] Poopak, B. et al. PCR analysis of IgH and TCR-gamma gene rearrangements as a confirmatory diagnostic tool for lymphoproliferative disorders. *Indian J. Hematol. Blood Transfus.* **31**(1), 38–45 (2015). 25548443 10.1007/s12288-014-0387-z PMC4275529

[37] Li, P., Cheung, L. & Chiu, B. Early bronchus-associated lymphoid tissue lymphoma diagnosed with immunoglobulin heavy chain molecular testing. *Can. Respir. J.* **2016**, 056035 (2016). 10.1155/2016/7056035 PMC4904558 27445561

[38] Torlakovic, E. et al. B-cell gene rearrangement in benign and malignant lymphoid proliferations of mucosa-associated lymphoid tissue and lymph nodes. *Hum. Pathol.* **28**(2), 166–173 (1997). 9023397 10.1016/s0046-8177(97)90101-5

[39] Hsieh, C. Y. et al. Nasal dissemination of a single-clone IgH-rearranged conjunctival MALT lymphoma through the nasolacrimal duct: A case report. *Oncol. Lett.* **12**(2), 1007–1010 (2016). 27446385 10.3892/ol.2016.4700 PMC4950472

[40] Ding, B., Ju, Z. C. & Liu, J. M. A correlation study between IgH gene rearrangement and orbital lymphoma removal operation prognosis. *Eur. Rev. Med. Pharmacol. Sci.* **21**(11), 2561–2566 (2017). 28678330

